# 25. Relative Effectiveness of Adjuvanted Trivalent Influenza Vaccine Compared to Egg-Based Trivalent High-Dose Influenza Vaccine among U.S. Older Adults during 2019-20 Influenza Season

**DOI:** 10.1093/ofid/ofab466.227

**Published:** 2021-12-04

**Authors:** myron J levin, Victoria Divino, Stephen I Pelton, Maarten Postma, Drishti Shah, Joaquin F Mould-Quevedo, Mitchell DeKoven

**Affiliations:** 1 University of Colorado Anschutz Medical Campus, Aurora, Colorado; 2 IQVIA, Falls Church, Virginia; 3 Boston Medical Center, Boston, Massachusetts; 4 University of Groningen, Groningen, Groningen, Netherlands; 5 Seqirus Vaccines Ltd., Summit, NJ

## Abstract

**Background:**

According to the Centers for Disease Control and Prevention (CDC), during the 2019-20 U.S. influenza season, influenza resulted in almost 180,000 hospitalizations and over 13,000 deaths in adults ≥ 65 years. The current study evaluated the relative vaccine effectiveness (rVE) of adjuvanted trivalent influenza vaccine (aTIV) compared to high-dose trivalent influenza vaccine (TIV-HD), against influenza-related hospitalizations/emergency room (ER) visits, all-cause hospitalizations and hospitalizations/ER visits for cardio-respiratory disease (CRD) among adults ≥65 years for the 2019-20 influenza season.

**Methods:**

A retrospective cohort analysis of older adults (≥ 65 years) was conducted using IQVIA’s professional fee, prescription claims and hospital charge master data in the U.S. Baseline characteristics included age, gender, payer type, geographic region, Charlson Comorbidity Index (CCI), comorbidities, indicators of frail health status, and pre-index hospitalization rates. To avoid any influenza outcome misclassification with COVID-19 infection, the study period ended March 7, 2020. Adjusted analyses were conducted through inverse probability of treatment weighting (IPTW) to control for selection bias. Poisson regression was used to estimate the adjusted pairwise rVE against influenza-related hospitalizations/ER visits, all-cause hospitalizations and any hospitalization/ER visit for CRD. An unrelated negative control outcome, urinary tract infection (UTI) hospitalization was included.

**Results:**

During the 2019-20 influenza season, following IPTW, 798,987 recipients of aTIV and 1,655,979 recipients of TIV-HD were identified. After IPTW adjustment and Poisson regression, aTIV was statistically comparable to TIV-HD for prevention of influenza-related hospitalizations/ER visits (3.1%; 95% CI: -2.8%-8.6%) and all-cause hospitalizations (-0.7%; 95% CI: -1.6%-0.3%). Similar comparable outcomes were found for reduction of any hospitalization/ER visit for CRD (0.9%; 95% CI: 0.0%-1.7%). No treatment effect was identified for the negative control outcome.

**Conclusion:**

aTIV and TIV-HD demonstrated comparable reductions in influenza-related hospitalizations/ER visits, all-cause hospitalizations and hospitalizations/ER visits for CRD.

**Disclosures:**

**myron J. levin, MD**, **GSK group of companies** (Employee, Research Grant or Support) **Victoria Divino, PhD**, **Seqirus** (Consultant) **Stephen I. Pelton, MD**, **Seqirus** (Consultant) **Maarten Postma, Dr.**, **Seqirus** (Consultant) **Drishti Shah, PhD**, **Seqirus** (Consultant) **Joaquin F. Mould-Quevedo, PhD**, **Seqirus** (Employee) **Mitchell DeKoven, PhD**, **Seqirus** (Consultant)

